# Protein Kinase CK2: Intricate Relationships within Regulatory Cellular Networks

**DOI:** 10.3390/ph10010027

**Published:** 2017-03-05

**Authors:** Teresa Nuñez de Villavicencio-Diaz, Adam J. Rabalski, David W. Litchfield

**Affiliations:** 1 Department of Biochemistry, Schulich School of Medicine & Dentistry, University of Western Ontario, London, ON N6A 5C1, Canada; tnunezde@uwo.ca (T.N.d.V.D.); arabalsk@uwo.ca (A.J.R.); 2 Department of Oncology, Schulich School of Medicine & Dentistry, University of Western Ontario, London, ON N6A 5C1, Canada

**Keywords:** protein kinase CK2, post-translational modification, regulatory networks, protein–protein interaction networks, hierarchical phosphorylation, post-translational modification interplay

## Abstract

Protein kinase CK2 is a small family of protein kinases that has been implicated in an expanding array of biological processes. While it is widely accepted that CK2 is a regulatory participant in a multitude of fundamental cellular processes, CK2 is often considered to be a constitutively active enzyme which raises questions about how it can be a regulatory participant in intricately controlled cellular processes. To resolve this apparent paradox, we have performed a systematic analysis of the published literature using text mining as well as mining of proteomic databases together with computational assembly of networks that involve CK2. These analyses reinforce the notion that CK2 is involved in a broad variety of biological processes and also reveal an extensive interplay between CK2 phosphorylation and other post-translational modifications. The interplay between CK2 and other post-translational modifications suggests that CK2 does have intricate roles in orchestrating cellular events. In this respect, phosphorylation of specific substrates by CK2 could be regulated by other post-translational modifications and CK2 could also have roles in modulating other post-translational modifications. Collectively, these observations suggest that the actions of CK2 are precisely coordinated with other constituents of regulatory cellular networks.

## 1. Introduction

Since its original discovery more than 50 years ago, protein kinase CK2 has been implicated in a continually expanding array of biological processes [1]. In this respect, CK2 has been shown to be a participant in the regulation of cellular processes such as transcription [2,3] and translation [4,5,6,7], control of protein stability [8,9,10] and degradation [11,12], cell cycle progression [13], cell survival [14,15,16], and circadian rhythms [17]. CK2 has also been linked to various aspects of tumor progression and suppression and has been shown to be elevated in many forms of cancer [18,19] as well as in virally infected cells [20,21,22]. Consequently, CK2 has recently emerged as a potential therapeutic target with two CK2 inhibitors, namely CX-4945 [23,24] and CIGB-300 [25,26], currently in clinical trials [27,28,29] for cancer treatment.

In humans, CK2 is typically considered to be a tetrameric enzyme comprised of two catalytic subunits (CK2α and/or CK2α′ subunits that are encoded by the CSNK2A1 and CSNK2A2 genes, respectively) and two regulatory CK2β subunits [1,30] (encoded by the CSNK2B gene). Although typically classified as a protein serine/threonine kinase based on sequence relationships to other members of the protein kinase superfamily, CK2 has also been shown to exhibit protein tyrosine kinase activity [31,32,33,34]. Biochemical characterization of its enzymatic activity has demonstrated that CK2 is an acidophilic kinase with a consensus recognition motif that features aspartic acid and glutamic acid residues as well as some phosphorylated residues as its dominant specificity determinants [35,36,37]. Characterization of its specificity determinants has contributed to the identification of many CK2 substrates that have been shown to be directly phosphorylated by CK2 [38,39]. Phosphoproteomic profiling has also revealed many putative substrates that have been shown to be phosphorylated in cells at sites that match the consensus for phosphorylation by CK2 [40,41]. In fact, analysis of phosphoproteomic datasets typically suggests that CK2 could be responsible for more than 10% of the phosphoproteome [42].

While there is ample evidence that CK2 is an important constituent in the regulation of many fundamental biological processes, there are unresolved issues regarding its regulation in cells. In this respect, questions regarding its regulation arise because the catalytic subunits of CK2 are enzymatically active in the presence or absence of the regulatory CK2β subunit. Furthermore, the activity of CK2 is generally unaffected by second messengers, and unlike many kinases that are regulated by phosphorylation within an activation loop, the activation loop of CK2 is devoid of regulatory phosphorylation sites [43,44]. The fact that the catalytic subunits of CK2 are fully active when expressed as recombinant proteins in bacteria further suggests that the enzyme may be constitutively active [45,46,47]. Consequently, it is unclear how a constitutively active enzyme can be a key regulatory participant in tightly regulated cellular processes. To investigate this apparent paradox, we have examined the relationship between CK2 and other constituents of the regulatory networks within cells by performing text mining of the published literature and mining of proteomic databases. Furthermore, motivated by our demonstration that CK2 phosphorylation sites overlap with other post-translational modifications to enable CK2 to modulate caspase cleavage [48,49,50] and to participate in hierarchical phosphorylation relationships, we have analyzed proteomic databases to identify post-translational modifications that may regulate, or be regulated by, CK2 phosphorylation. 

In this review, we explore database and literature information available in the context of CK2-dependent signaling with the objective of highlighting and discussing the extensive interplay of CK2 with regulatory networks in the cell. The analysis that we have provided is also intended to provide functional perspectives to the data available in the databases since these data are often isolated from the information regarding CK2 that exists within the literature; especially for non-experts in the field and for interpreting data related to CK2 emerging from high-throughput studies.

## 2. CK2 Networks

### 2.1. Functional Networks Involving CK2

The impact of CK2 in the cell can be, to some extent, predicted by considering the number of biological processes in which it has been implicated and the number of substrates that have been reported to date [39]. Furthermore, it is anticipated that the published literature will represent an important resource for deciphering and validating information that is emerging from genome- and proteome-wide analyses. Consequently, we performed text mining to identify publications that highlight CK2 and aspects of its function or regulation. To this point, CK2 substrate information remains “sparse” in the published literature that is represented by more than 2600 papers (PubMed [51] search: “Casein Kinase II” [Mesh]) directly describing CK2 function and more than 5000 papers mentioning the kinase (GoPubMed [52] search). Nevertheless, assembling this information to obtain a global but detailed view of CK2-dependent networks is one step towards deciphering genome and proteome scale analyses of CK2.

**Figure 1:**
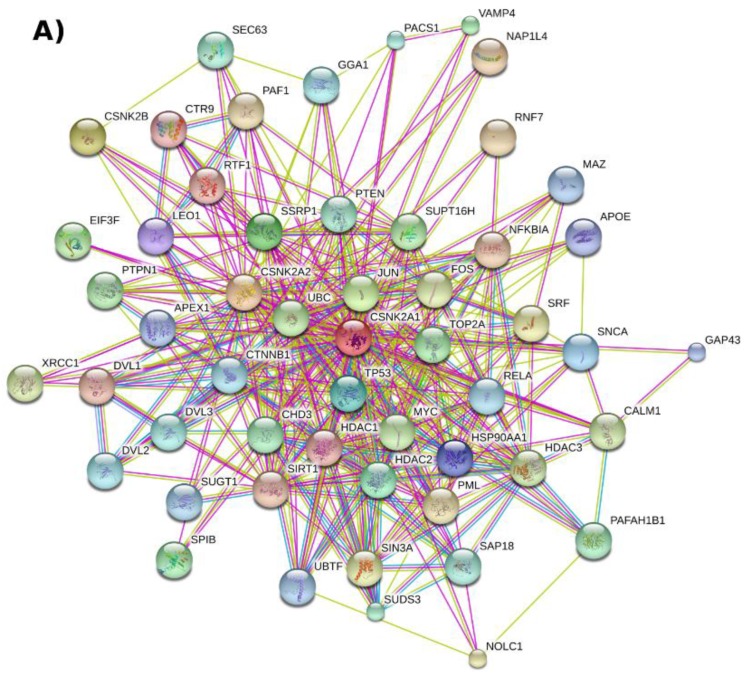

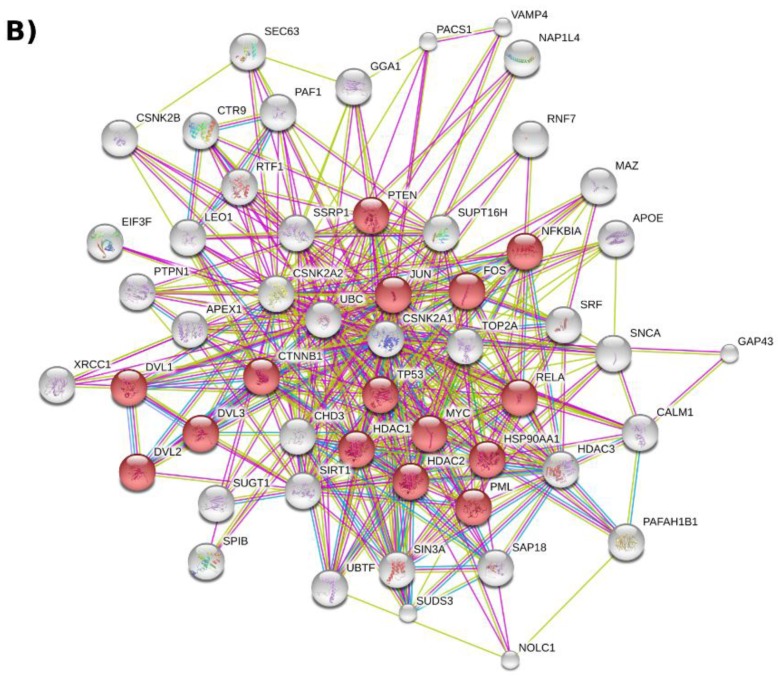

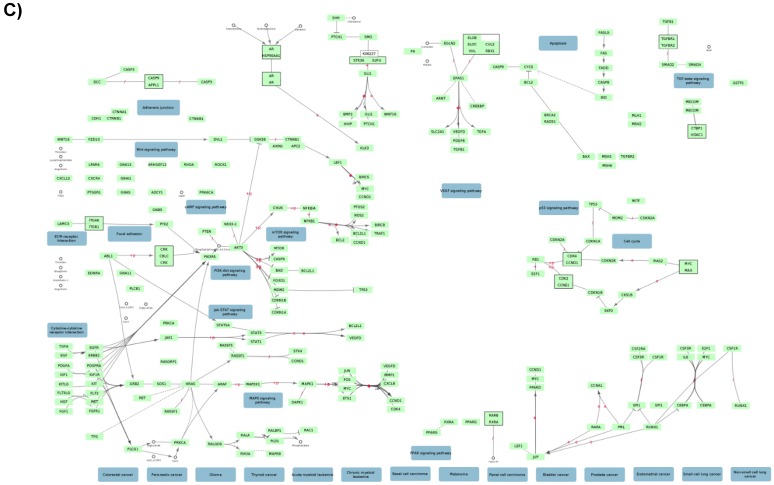
CK2-centered functional association network represented with the database STRING v10.0. (**A**) The network shows the top 50 human genes related to CK2 subunits CSNK2A1, CSNK2A2, and CSNK2B (number of nodes and edges: 53 and 369, respectively) based on neighborhood, experiments, text mining, and database sources. Briefly, the human CK2 subunits were searched in STRING by gene name using the multiple protein search functionality. The functional association network was then retrieved by selecting 50 as the maximum number of interactors to show from the first shell (step 1) and checking the mentioned interaction sources (these options are found within the “data settings” drop down menu); (**B**) A clone of network A with the red nodes representing proteins connected to deregulated pathways (KEGG pathway: hsa05200); (**C**) A network representation of the map: Pathways in cancer (KEGG pathway: hsa05200). The map was downloaded from the KEGG PATHWAY [63] database (accessed 30 November 2016) and imported to Cytoscape with the CyKEGGParser v1.2.7 plugin [64]. A high-resolution image of this figure is also available in the Supplementary Materials.

An initial evaluation of the functional relationship of CK2 with other cellular proteins can be obtained by querying STRING v10.0 [53], a database with known and predicted functional associations between proteins (see Figure 1A for a representation of the top 50 proteins functionally associated to CK2, Table S1). According to this analysis, CK2 regulates the activity of at least 15 cancer-related proteins such as the tumor suppressor TP53, the histone deacetylases HDAC1 and HDAC2, and the NFKB subunit RELA (Figure 1B,C, Table S1). CK2-dependent phosphorylation of these proteins has been reported either in vitro or in vivo [54,55,56,57] with the majority of the target sites identified conforming to the minimum CK2 consensus sequence: [ST]xx[DEpS]. The CK2 functional relationship to these substrates places the kinase in a central position in human protein–protein interaction networks since such proteins are considered “information hubs” [58]. For instance, the proteins TP53, HDAC1, HDAC2, and RELA bind to at least 997, 554, 323, and 271 unique interactors based on the BioGRID protein–protein interaction repository [59] (accessed 30 November 2016). In fact, in a human protein–protein interaction network (built from the BioGRID database in Cytoscape [60,61] v3.4.0, self-loops and duplicated edges removed) CK2 can “influence” approximately 23% of the established interactions (63,988 interaction pairs out of 270,000) if we assume a ‘guilt by association’ approach considering CK2 direct interactions (meaning step 1 interactors: 629 proteins, Table S2) and that of its indirect interactors (meaning step 2 interactors: 11,869 proteins, Table S2). A summary of the number of CK2 interactors for each human CK2 subunit is presented in Table 1. Based on this analysis and a search using the “find a gene” functionality of the Enrichr tool [62], further hub interactors of CK2 that can be identified include CDK1, XRCC6, CREB1, HNRNPA1, LYN, YWHAQ, FOS, and MAPK1 (Table S3).

### 2.2. Protein–Protein Interaction Networks Involving CK2

**Figure 2:**
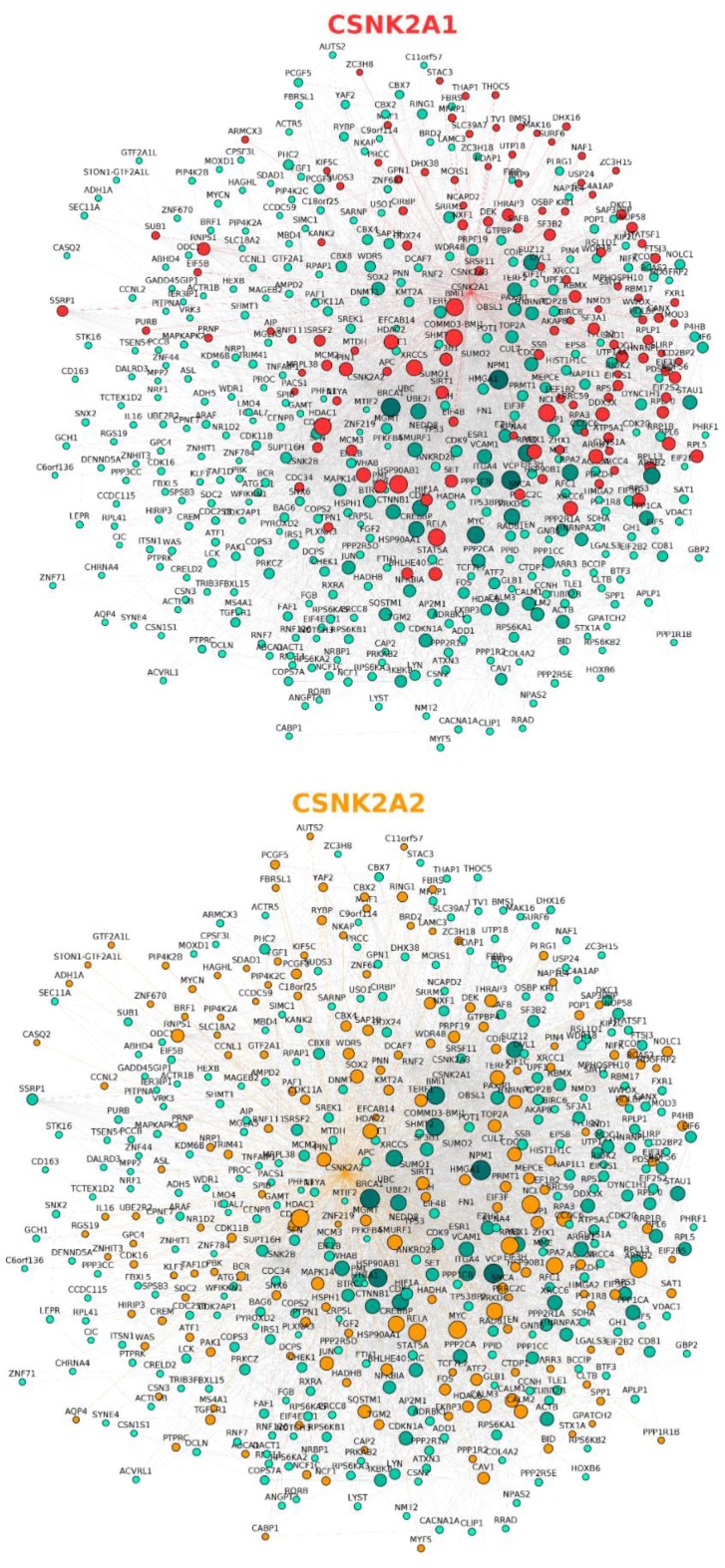

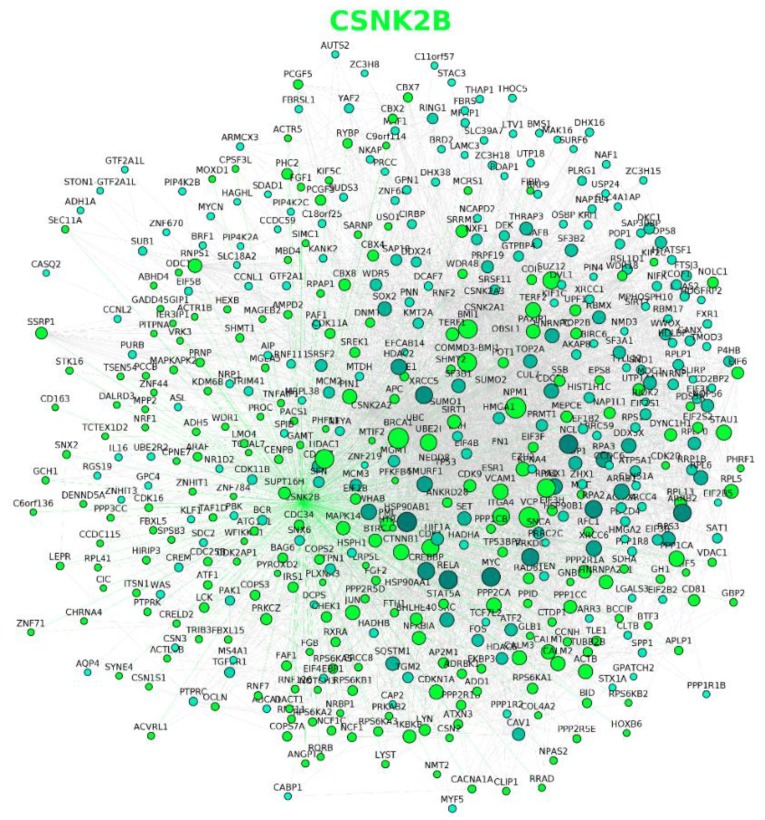
Protein–protein interaction network of human CK2 subunits generated using Cytoscape v3.4.0 and the BisoGenet v3.0.0 plugin. The three networks are clones, each of which highlights the interactors of CSNK2A1 (red nodes), CSNK2A2 (orange nodes), and CSNK2B (green nodes) subunits, respectively. Briefly, the network was represented by querying SysBiomics (BisoGenet’s interaction database) through the plugin’s interface using the human CK2 subunit gene names and selecting “protein–protein interaction” as the biorelation type and the input nodes and neighbors to step 1 method as the criteria for building the network. A high resolution image of this figure is also available in the Supplementary Materials.

In a complementary analysis, the direct interactors of CK2 subunits were represented in a protein–protein interaction network (Figure 2, Table S4) using BisoGenet [65] v3.0.0 Cytoscape plugin for the retrieval of physical interaction information. As expected from Table 1, differences in the number and identity of the panel of interactors can be observed for each CK2 subunit which suggests a certain degree of functional divergence as previously highlighted in the literature [66,67,68,69,70], and encourages the development of tools that allow us to differentiate the contributions of the endogenous catalytic subunits to the phosphoproteome. In addition, it points to CSNK2B as a hub itself, suggesting that it may have a role in coordinating interactions with the catalytic subunits of CK2 to modulate phosphorylation of certain substrates. Furthermore, since the interaction network for CSNK2B does not completely overlap that of the catalytic CK2 subunits (Figure 2, Table S4), this analysis reinforces the prospect that CSNK2B has CK2-independent roles within cells [67]. In fact, the CSNK2B-dependent interactome has been previously profiled using mouse brain homogenates [71] where CSNK2B is thought to have a crucial role since its mRNA expression levels are 2–3-fold higher compared with other organs, except the testis [72]. In this setting, CSNK2B was found to interact with both cytoplasmic and nuclear localized proteins involved in protein synthesis, RNA and DNA processing, the cytoskeleton, cell signaling, and transport [71]. Although not included among the references retrieved by BisoGenet, the functional classification of the proteins identified as part of the CSNK2B-dependent interactome is in agreement with the network generated by BisoGenet.

### 2.3. CK2 Networks Derived from Text Mining of the Published Literature

**Figure 3:**
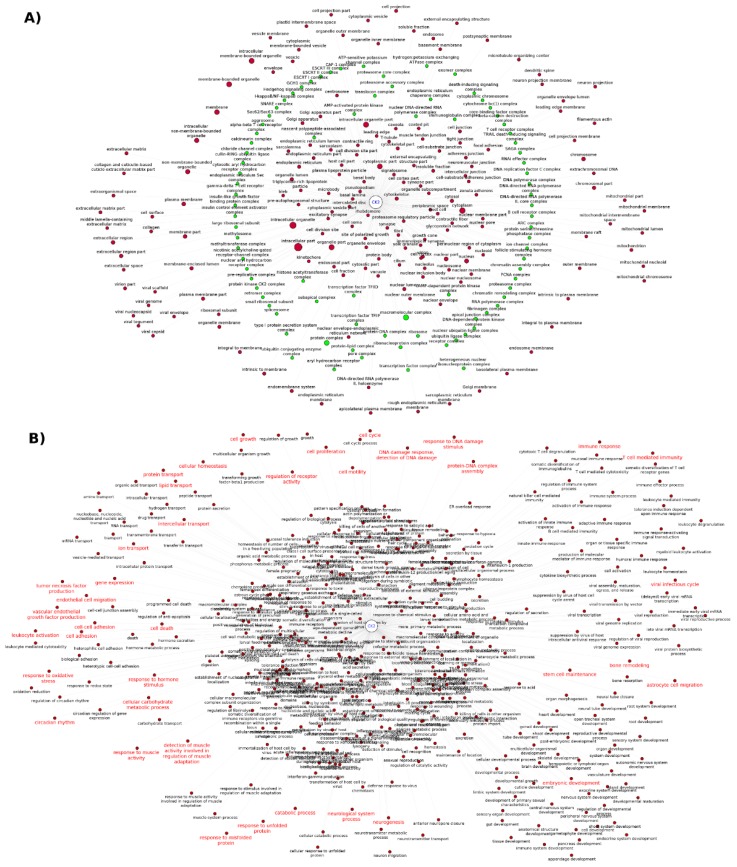
CK2 Functional association networks represented in Cytoscape v3.4.0 for illustrating the: (**A**) Cellular components mentioned together with CK2 in the literature identified by GoPubMed search. The green nodes represent protein complexes and the node size highlights terms frequently co-occurring with CK2; (**B**) Biological processes mentioned together with CK2 in the literature identified by GoPubMed search; the red label-nodes indicate hot topics in CK2 research. Briefly, the term “Casein Kinase II”[Mesh] was queried using the GoPubMed text mining tool and the output was downloaded to generate a network in SIF format [61] for Cytoscape input by specifying CK2 and the GO subcellular components/biological processes as nodes and their co-occurrence in the literature as the interactions (binary type: yes/no). The node size was not set differentially for the biological processes network as this will affect the visualization and readability. A high resolution image of this figure is also available in the Supplementary Materials.

A way of summarizing and systematizing CK2 knowledge relies on the use of text mining to access literature information. In this regard, the analysis of GO cellular component annotations using GoPubMed revealed more than 200 subcellular locations and protein complexes studied in the context of CK2 (summarized in Figure 3, Table S5). This analysis also reflects the functional pleiotropy of CK2, which can associate with molecular machinery such as the ribosome, spliceosome, proteasome, and chromatin remodeling complexes, and to other smaller more dynamic complexes such as TRAIL-death inducing complex and the Ikappa-NFkB complex. Directly interrogating the literature followed by data extraction can provide information that otherwise may be missed if we only consider specialized databases such as the mammalian protein complexes database CORUM [73] where CSNK2A1, CSNK2A2 and CSNK2B (Table S5) are listed only as members of the “PDGF treated Ksr1-CK2-MEK-14-3-3 complex”, the “MKP3-CK2alpha complex”, the “Casein kinase II-HMG1 complex”, and the “UV-activated FACT complex” with CSNK2B also listed as a member of the “Fgf2-Ck2 complex” (accessed 30 November 2016). However, protein complex data extracted from the CORUM database has been manually curated whereas the data extracted from the literature needs to be critically analyzed since the extraction process may be ambiguous and biased towards the algorithm used by the tool [74], in this case GoPubMed. A similar analysis can be made for the GO biological processes. The retrieval of CK2 related GO biological process from literature highlights frequently studied core processes such as cell cycle, cell proliferation, DNA damage, cell death, and viral infectious cycle, as well as other hot topics in the field (Figure 3B, Table S5). The later includes the involvement of CK2 in embryogenic development [75,76,77,78], T cell-mediated immunity [79,80], inflammation [81], glucose homeostasis [82,83,84,85], ion transport [86], bone remodeling [78,87], neurogenesis [88,89], neurological system process [90], response to misfolded and unfolded protein [90], stem cell differentiation and maintenance [91], and response to muscle activity [92,93].

Text mining of the CK2-related literature can also provide insights regarding holoenzyme-dependent regulation, which relates to the events where CK2-mediated phosphorylation of a given substrate is positively or negatively regulated by holoenzyme formation and the presence or absence of CSNK2B [45]. In this particular case, the analysis of protein–protein interaction data for CK2 subunits alone is insufficient for assuming holoenzyme-dependent regulation. A recent in silico study relied on text mining for retrieving known holoenzyme-dependent substrates and generated sequence patterns for predicting novel candidates based on structural information of the known substrates [39]. Information on the holoenzyme-dependent substrates can also be obtained from PhosphoSitePlus database [94,95] by querying “substrates of CK2B”; however the list obtained is not comprehensive (accessed February, 2017) when compared to the text mining study [39]. Furthermore, substrates known to be holoenzyme-dependent such as PDX1 [96] and CFTR [97] are cataloged as phosphorylated by the catalytic subunit in this database (query: “substrates of CK2A1”). To avoid such inconsistencies and misleading information, researchers are encouraged to carefully review the evidence provided in databases such as PhosphoSitePlus, which are obtained through automated literature text mining and thus error prone.

In addition to text mining, systematic proteomic studies represent rich resources for potentially uncovering CK2-regulated biological processes and pathways when datasets from these studies are uploaded to repositories [98] such as PRIDE and MassIVE and/or provided as supplementary information. To explore the availability of proteomics data for CK2, we performed a Pubmed search and, as a result, retrieved two proteome studies [99,100], one interactome study [71], and four phosphoproteome [40,41,101,102] studies. However, a PRIDE search only returned one of these studies, which explores the function of CSNK2A1 from *Ostreococcus tauri* in a minimal circadian system [101] (ID: PXD000975). Consequently, for the remainder of these studies, information available (e.g., protein and/or phosphorylation site identification and/or relative quantification values) is limited to that provided in the original paper or by direct request to the authors. We also searched PhosphoSitePlus and found that only one phosphoproteome study out of the four identified in Pubmed was included (accessed February 2017). This phophoproteomic study explores the short-term response of HEK-293T cells treated with the CK2 inhibitor quinalizarin [102]. However, PhosphoSitePlus only mentions the proteins and phosphosites identified without providing any quantitative results.

The quinalizarin phosphoproteomic study [102] identified 28 downregulated putative CK2 phosphosites with several of the target proteins displaying a role in cell death and/or survival including TPD52 (isoform 2), STX12, BCLAF1, AKAP12, RAD50, and PDCD4 (isoform 2). Overall, the majority of the phosphorylation-modulated substrates were classified as nuclear and were found to be involved in biological processes classified as transcription, mRNA and rRNA processing, gene expression, and DNA replication. Comparable functional annotations were obtained in two of the other CK2 phosphoproteomic studies where the phosphosites identified belong to proteins mostly localized to the nucleus as components of the spliceosome [40,41].

Intriguingly, the quinalizarin phosphoproteomic study also revealed that several “CK2 attributable” phosphosites increased upon treatment with the inhibitor. As this result seems paradoxical, the authors proposed both technical and biological explanations for this observation [102]. Since it is evident that CK2 is connected to a plethora of regulatory hubs through protein–protein interaction and hierarchical phosphorylation, we performed a kinase-motif matching analysis using the PhosphoMotif Finder functionality of the HPRD database [103] to determine if other kinases could in theory be responsible for the upregulation of phosphosites that had been putatively identified as CK2-dependent phosphosites. As a result of this analysis, we identified at least two instances where the modulated phosphosite matched motifs for other kinases besides CK2. For example, the vicinity of the residue S1068 of TP53BP1 matches the minimal CK2 consensus sequence pSXX[E/D] as well as the pSQ and XpSQ substrate motifs of the ATM kinase and the DNA-dependent protein kinase, respectively. Interestingly, TP53BP1 does interact with ATM, which phosphorylates several residues in the protein upon DNA damage to promote its tumor suppressor functions [104]. Another example is AKAP12 where the vicinity of S627 matches substrate recognition motifs pSXX[E/D], RXRXX[pS/pT], [R/K]XRXXpS, RVRRPpSESDK, and RRPpS conforming to CK2, AKT, MAPKAPK1, AMP-activated protein kinase 2, and PKA/PKC motifs, respectively. AKAP12 has been shown to interact at least with PKC and PKA [105], with PKA phosphorylating S627 and three other residues [106] of the protein. Moreover, in proteomic databases, arginine and lysine residues proximal to S627 of AKAP12, are reported to be methylated [98]. Altogether, these observations illustrate the complexity of CK2-signaling networks and the challenges associated with interpreting changes in the phosphoproteome arising from modulation of CK2. Accordingly, all possible sources of information for other kinases and modifying enzymes that may act upon CK2 target sequences (e.g., arginine-methyltransferases and lysine-acetyltransferases) need to be taken into consideration.

### 2.4. Extension of CK2 Networks to Include Other Constituents of Regulatory Networks

**Figure 4:**
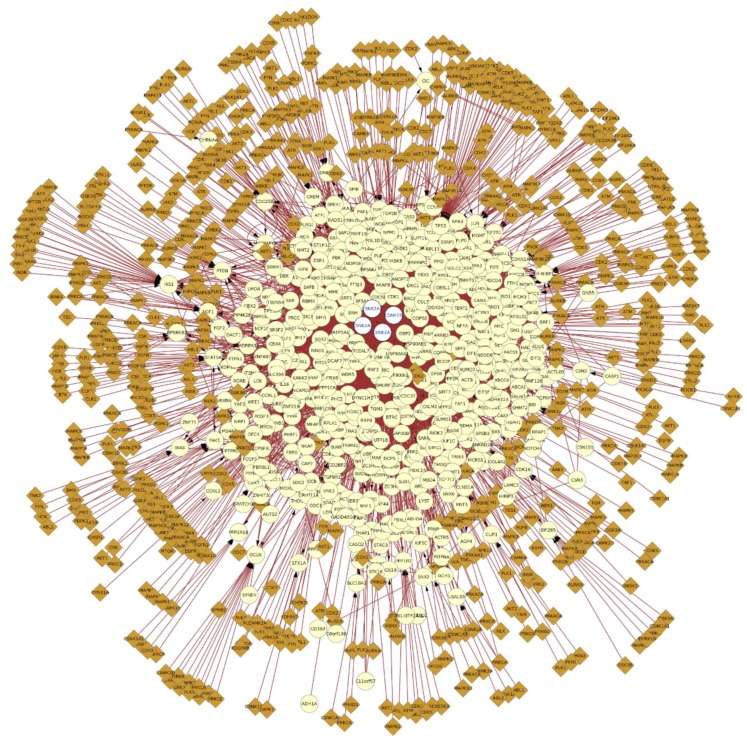
Protein–protein interaction network of CK2 subunits expanded to include kinase information of the interactors retrieved from PhosphositePlus, the network was represented with Cytoscape v3.4.0 using the BisoGenet v3.0.0 plugin and the PhosphositePlus Web Service Client Module. The yellow nodes represent CK2 interactors and the brown nodes represent kinases that phosphorylate (edges with arrow) the interactors. The blue edges represent protein–protein interactions and the red edges represent phosphorylation events. The network was represented as explained in Figure 2. Briefly, all the proteins were selected and the kinase data was added by importing the information from PhosphoSitePlus using the plugin and selecting the gene name as the matching key. A high resolution image of this figure is also available in the Supplementary Materials.

As a logical extension of examining direct interactions with CK2, understanding how CK2 integrates to other signaling networks in the cell requires consideration of how CK2 substrates may be acted upon by other constituents of signaling networks. For example, we have considered phosphorylation information regarding CK2 substrates with other kinases that also modify the CK2 substrates and/or interactors (Figure 4). In this respect, Figure 4 shows that at least 171 other kinases (Table S6) are capable of phosphorylating sites in CK2 interactors, which suggests a likelihood of functional interplay among phosphorylation sites. This is reflected, for example, in the fact that CK2-dependent phosphorylation often participates in hierarchical phosphorylation with other kinases, which generates a CK2 target sequence with a phosphorylated serine that functions as the dominant specificity determinant [36].

As noted above, there is evidence that many CK2 substrates are also phosphorylated by other kinases. When considering the prospect for hierarchical phosphorylation involving CK2, analysis of the phosphorylation events in the vicinity of the CK2 phosphosites may further contribute to identifying such relationships. Consequently, we searched PhosphoSitePlus database serine, threonine, and tyrosine phosphorylation and CK2 substrate data, mostly reflecting data generated through a proteomics approach [95], for those phosphosites occurring in the primary structure at different distance windows up- or downstream of the CK2 target site (Table 2 and Table S6). This analysis was also extended to other post-translational modifications reported in the PhosphoSitePlus database, such as ubiquitination and acetylation (Table 2 and Table S6). Although this analysis is restricted to CK2 substrate data available in PhosphoSitePlus, it is useful to illustrate the importance of considering the modification status of target sequences when studying CK2-dependent phosphorylation, a concept that also applies to other kinases. For example, the link between AKT-mediated phosphorylation and arginine methylation has been previously reported [107,108,109]. The post-translational modification analysis indicated that the vicinity of CK2 sites may constitute ‘hot spots’ for phosphorylation. Consideration of these sites brings together at least 76 different kinases including CDK7, STK1, PRKCA, PLK1, GSK3, CDC7, SRC, CDK2, MAPK3, GRK2, PRKCZ, CDK9, MAPK1, and IKBKE.

## 3. Concluding Remarks and Implications

Taken together, systematic analysis of the literature and databases strongly reinforces the view that CK2 is involved in a broad spectrum of biological processes. At the same time, it is important to recognize that there are significant limitations with information that is available both within the peer-reviewed literature and in databases. In this respect, as noted earlier, the literature represents an extensive but ‘sparse’ body of information generally lacking standard criteria for the identification and characterization of roles for CK2 in biological processes. On the one hand, there are undoubtedly many literature reports with rigorous experimental design and analysis that clearly demonstrate roles for CK2 in specific cellular processes. By comparison, there are also reports where conclusions are drawn exclusively from in vitro studies or experimentally contrived systems that do not reflect what happens under normal physiological conditions in living systems. In a similar vein, databases that are populated primarily from large-scale proteomic studies or high-throughput experimental workflows harbor data of inconsistent integrity. While these databases represent a wealth of information (for example, tens of thousands of phosphorylation sites or protein–protein interactions), the pace at which data is generated dramatically exceeds the pace of validation. Moreover, many of the kinase-substrate relationships that have emerged from large-scale studies have relied on predictions using the CK2 consensus recognition motif rather than experimental validation of these relationships. Consequently, in many instances, these data reflect what ‘might happen’ rather than rigorous demonstrations of what ‘does happen’ under normal physiological conditions. Despite this limitation, knowing what ‘might happen’ can often be a very useful guide for the design of experimental strategies to rigorously define what does happen.

In addition to clearly highlighting the involvement of CK2 in a broad array of biological processes, database mining and network analysis clearly reveals extensive interplay between CK2 and other constituents of signaling networks. This interplay is evident from the identification of ‘hot spots’ where CK2 phosphorylation sites are localized proximal to other phosphorylation sites or other post-translational modifications within the primary sequence of its substrates. The proximity of post-translational modifications to one another raises the very interesting prospect of one post-translational modification being regulated by others. As noted earlier, we have previously demonstrated that CK2 can modulate cleavage of caspase substrates when CK2 phosphorylates residues adjacent to the cleavage site [48]. This is a clear example of how phosphorylation by CK2 can modulate susceptibility to another post-translational modification. The demonstration that phosphorylated residues can sometimes ‘prime’ a substrate for hierarchical phosphorylation by CK2 demonstrates that phosphorylation by CK2 can also be regulated by other modifications [36]. Considering the prevalence of post-translational modifications that reside within CK2 substrates within close proximity of the CK2 phosphorylation site (Table 2), it will be important to consider the relationship between CK2 phosphorylation and these other modifications. From the perspective of its participation in regulatory processes, the interplay between CK2 and other pathways could yield very intricate and precise control of processes. Considering the emergence of CK2 as a potential therapeutic target, the intricate relationships of CK2 within the regulatory networks also has important implications for the application of CK2 inhibitors. From this perspective, modulation of CK2 could impact other pathways since phosphorylation by CK2 could have regulatory consequences for other pathways. Similarly, modulation of other pathways could also affect CK2 or its functions as the modification status of the target sequence may ultimately regulate its recognition by CK2.

With the availability of selective new inhibitors for CK2 and elegant strategies for genome engineering based on CRISPR-Cas9, we can expect new opportunities for modulating CK2 under physiological conditions. By combining these strategies with striking advances in analytical technologies such as mass spectrometry to perform proteomic profiling with unrivalled depth, speed, and accuracy, we can anticipate that many gaps in our knowledge of how CK2 is integrated within regulatory and functional networks will be filled to reveal its roles in orchestrating biological processes. Furthermore, given the emergence of CK2 as a potential therapeutic target, we can expect these insights to guide promising applications of CK2 inhibitors in the clinic.

## Figures and Tables

**Table 1:** CK2 subunits interactors extracted from a human protein–protein interaction network built by retrieving the human interactome from BioGRID database build 3.4.129 using Cytoscape v3.4.0.

Direct (Step 1) Interactors of	Number of Interactors
CSNK2A1 (Gene ID: 1457)	398 unique direct interactors; 435 interaction pairs.
CSNK2A2 (Gene ID: 1459)	155 unique direct interactors; 171 interaction pairs.
CSNK2B (Gene ID: 1460)	247 unique direct interactors; 270 interaction pairs.
All CK2 subunits	632 unique direct interactors from which 36 are shared by the three subunits and 95 by two.
All CK2 subunits and their direct interactors	12,502 unique direct interactors (632 step 1 and 11,875 step 2); 63,988 interaction pairs.

**Table 2:** Post-translational modifications in the vicinity of CK2 ^1^ target sites retrieved from PhosphoSitePlus (accessed August 2016).

PTM Type	# Sites at −4/+4	# Sites at −7/+7	# Sites at −36/+36
Acetylation	15	25	146
*O*-*N*-acetylgalactosamine	2; overlap: 1	5; overlap: 1	19; overlap: 1
*O*-*N*-acetylglucosamine	1; overlap: 2	1; overlap: 2	5; overlap: 2
Methylation (m1, m2, m3, me)	4, 3, 1, none	8, 7, 1, none	29, 22, 2, 3
Phosphorylation	262; overlap: 482	395; overlap: 482	1177; overlap: 482
Sumoylation	1	8	50

^1^ CK2 sites were retrieved for bovine CSNK2A1 (P68399), human CSNK2A1 (P68400), human CSNK2A2 (P19784), human CSNK2B (P67870), mouse CSNK2A1 (Q60737), rat CSNK2A1 (P19139).

## Supplementary Materials

The following are available online at http://www.mdpi.com/1424-8247/10/1/27/s1; Table S1: STRING network, function, and annotations of the top 50 CK2-related genes; Table S2: CK2-interacting proteins in the human interactome; Table S3: CK2-interacting hub proteins; Table S4: CK2-interacting proteins of each subunit; Table S5: CK2-related GO annotations extracted from the literature and protein complex data; Table S6: PTM sites in the vicinity of CK2 phosphorylated sites.
