# Supplementary figures and images for: Protein Kinase CK2: Intricate Relationships within Regulatory Cellular Networks
Source: Pharmaceuticals (Basel). 2017 Mar 5;10(1):27. doi: 10.3390/ph10010027 (PMC5374431; doi:10.3390/ph10010027)

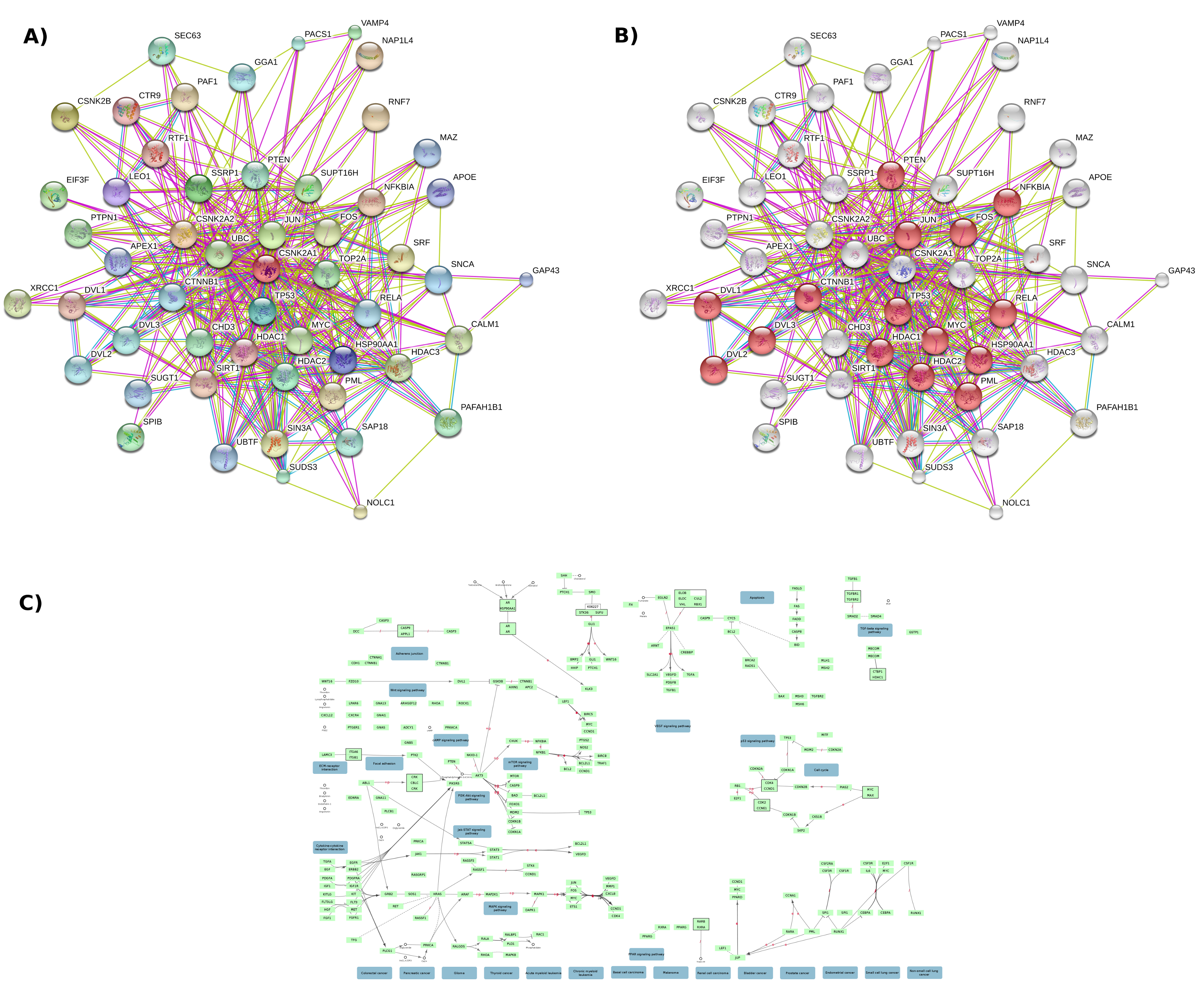

Supplement: Supplementary file 1 [file pharmaceuticals-10-00027-s001.zip › Supplementary Files/Figure1 (high resolution).png]

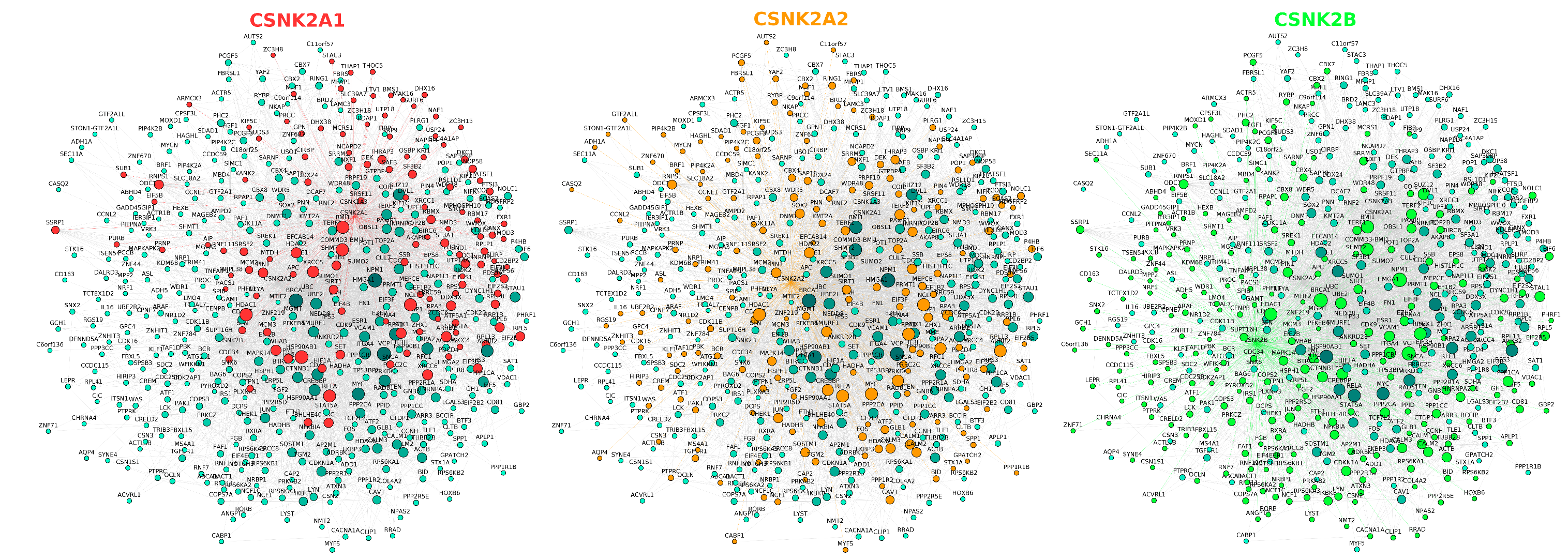

Supplement: Supplementary file 1 [file pharmaceuticals-10-00027-s001.zip › Supplementary Files/Figure2 (high resolution).png]

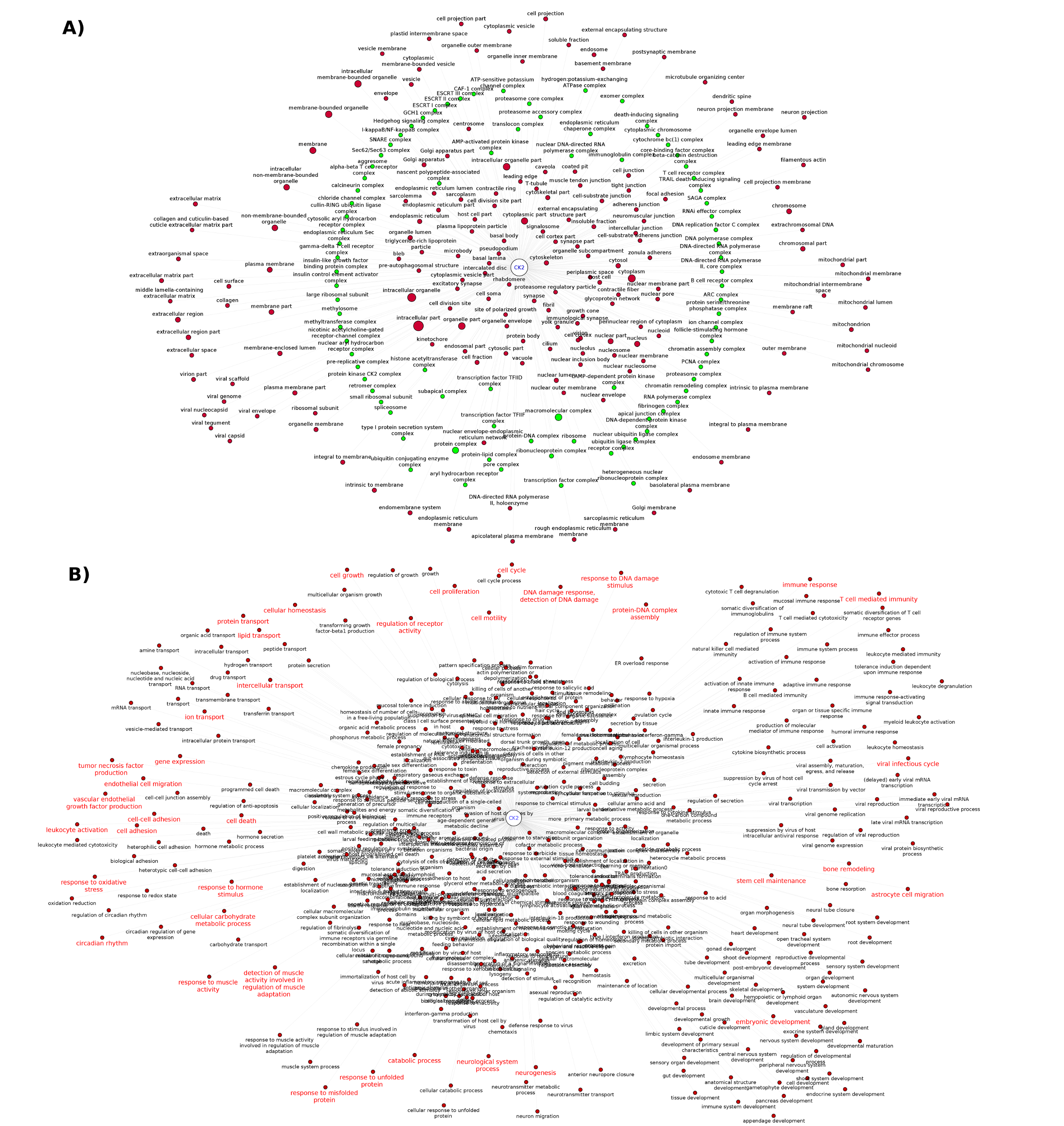

Supplement: Supplementary file 1 [file pharmaceuticals-10-00027-s001.zip › Supplementary Files/Figure3 (high resolution).png]

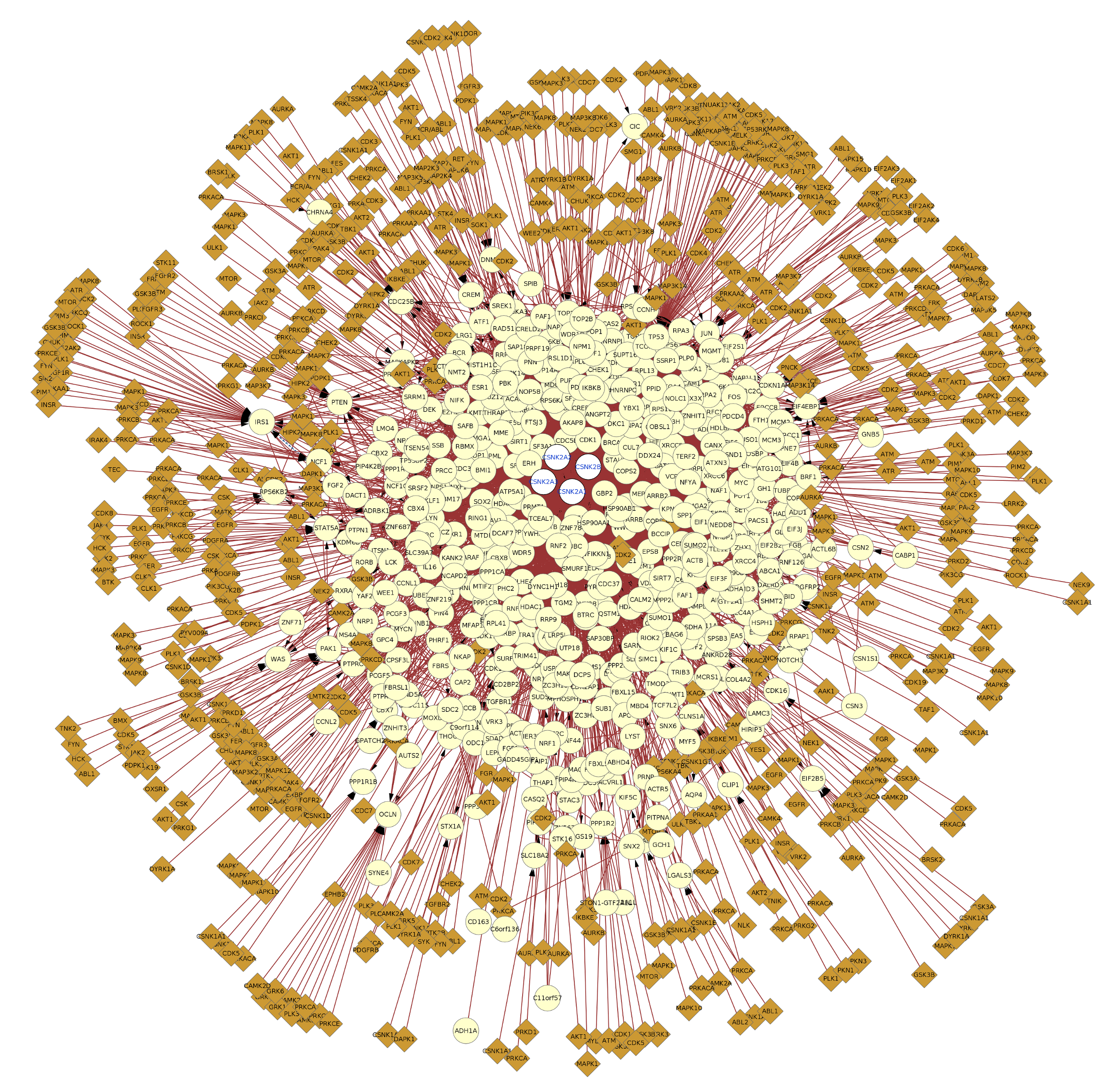

Supplement: Supplementary file 1 [file pharmaceuticals-10-00027-s001.zip › Supplementary Files/Figure4 (high resolution).png]
